# Core outcome sets and trials of nutrition and metabolism interventions

**DOI:** 10.1097/MCO.0000000000001191

**Published:** 2025-11-27

**Authors:** Annika Reintam Blaser, Thomas Davies, Kaspar F. Bachmann

**Affiliations:** a Department of Intensive Care Medicine, Lucerne Cantonal Hospital, Lucerne; b Department of Anaesthesiology and Intensive Care, University of Tartu, Tartu, Estonia, Switzerland; c William Harvey Research Institute, Barts and The London School of Medicine & Dentistry, Queen Mary University of London; d Adult Critical Care Unit, Royal London Hospital, London, UK; e Intensive Care Unit, Department of Acute Medicine, University Hospital Basel, Basel, Switzerland

**Keywords:** biomarkers, core outcome sets, critical care nutrition, physical function, postintensive care syndrome

## Abstract

**Purpose of review:**

The target of critical care nutrition research is moving from short-term physiological surrogate endpoints and mortality toward long-term patient-centered outcomes. This review summarizes recent core outcome set (COS) initiatives relevant to nutrition and metabolism and outcome selection in recent trials.

**Recent findings:**

The Core Outcome Measures for Clinical Effectiveness Trials of Nutritional and Metabolic Interventions in Critical Illness (CONCISE) defined essential outcomes: survival, physical function, infection, activities of daily living, nutritional status, and muscle/nerve function to be assessed at 30 and 90 days after randomization, with suggested but nonmandated instruments to preserve feasibility. COSMOGI (core outcome set of daily monitoring of gastrointestinal function in critically ill patients) standardizes daily gastrointestinal monitoring during critical illness. Large, randomized trials testing higher protein or early aggressive energy delivery have not improved survival and functional recovery, although the latter has only recently received more attention. From a mechanistic perspective, outcome selection in critical care nutrition and gastrointestinal function research should prioritize patient-centered (i.e. functional and patient-reported) outcomes.

**Summary:**

Standardizing outcome selection should improve interpretability and evidence synthesis. Future trials should incorporate robust functional and patient-reported outcomes. Core outcome sets will need updates when new assessment tools (i.e., biomarkers, new functional tests, standardized ultrasound protocols) emerge.

## INTRODUCTION

Critical care nutrition research has evolved in recent years, primarily due to a better understanding that surviving critical illness marks only the onset of a complex recovery trajectory [1^▪▪^]. As mortality rates decline, there is an increasing need to evaluate functional outcomes and quality of life measures that may better reflect the patient's status following hospital discharge. This shift has resulted in the development of standardized outcome measurement tools and prompted a reexamination of conventional nutritional interventions [2].

Emerging evidence suggests that minor, short-term alterations in nutritional interventions rarely result in substantial impacts on mortality due to low biological plausibility. However, depending on the timing and dose, these modifications may enhance physical function and quality of life for survivors, which are therefore important outcomes worth measuring. This paradigm shift necessitates robust, standardized measurement approaches that enable comparison across studies when assessing patient-centered outcomes. Standardized terminology and outcomes should also facilitate the development of consensus definitions for poorly defined entities such as gastrointestinal (GI) dysfunction, nonocclusive mesenteric ischemia, or GI paralysis [3]. In this review, we summarize recent advances in core outcome set development and highlight key findings from critical care nutrition and metabolism trials with an emphasis on outcome assessment. 

**Box 1:**
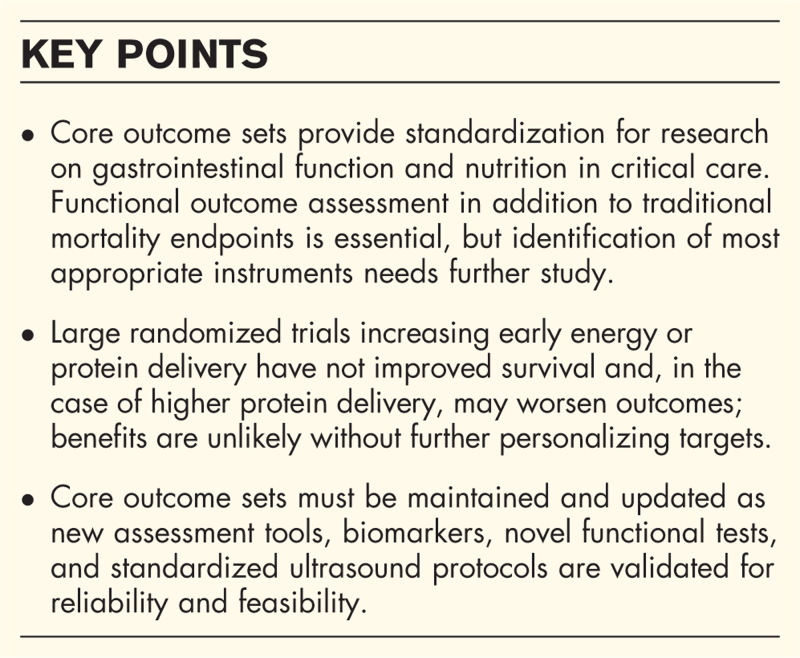
no caption available

## RECENT DEVELOPMENTS IN CORE OUTCOME SETS

Core outcome sets are agreed, standardized sets of outcomes that should be measured and reported, as a minimum, in all clinical trials for a specific area of health or healthcare. Their development involves diverse stakeholders, including patients and caregivers, clinicians, trialists, regulators, researchers, and policymakers, and follows established guidance provided by the COMET (Core Outcome Measures in Effectiveness Trials Initiative) [4]. A typical process includes identifying outcomes via systematic reviews and stakeholder input, followed by structured, anonymous Delphi surveys and consensus meetings to reach agreement on which outcomes should be included. Core outcome sets are intended to improve the relevance and comparability of trials, support evidence synthesis, reduce outcome reporting bias, and minimise research waste.

Core outcome sets (COS) are currently being developed for different aspects of critical care research [5,6^▪▪^–8^▪▪^,^9^]. In the past, large clinical trials in critical care nutrition research have focused on short-term interventions with mortality as the primary outcome. Negative results of these trials have contributed to a shift in understanding that functional patient-centered outcomes should be assessed [10,11^▪▪^,^12^]. However, long-term functional outcomes were often not evaluated as primary endpoints or were assessed only in secondary investigations that frequently did not include the entire intention-to-treat population. Functional outcomes have also been applied inconsistently, with significant variation in their definitions, timing, and measurement tools across studies, limiting comparability and interpretability of results [13^▪^]. Additionally, GI function has been largely overlooked as an important factor in nutrition studies, despite its impact on the efficacy of enteral nutrition delivery, the potential for life-threatening complications related to enteral nutrition, and its influence on mortality [15,45]. GI dysfunction affects patient comfort and well being during critical illness and may influence long-term morbidity and mortality [14^▪▪^]. In response, core outcome sets have been developed to provide a standardized approach to monitoring and outcome selection [16,17].

CONCISE defined the minimum set of outcomes for clinical effectiveness trials of nutritional and metabolic interventions in critical illness at 30 and 90 days postrandomization [16]. Selecting appropriate time points for outcome measurement is challenging in nutritional and metabolic intervention trials due to the wide variability in treatment duration and physiological response. Using fixed time points from randomization may increase study consistency and improve external validity. However, assessing outcomes beyond hospital stay is logistically more demanding for the involved study team. Stakeholders were recruited internationally from 24 countries, including patients who had survived critical illness or their caregivers, critical care clinicians, and clinical researchers interested in metabolic and nutritional interventions. The “essential” outcomes to include were survival, physical function, infection, activities of daily living (ADLs), nutritional status, and muscle/nerve function. Additional “recommended” outcomes included organ dysfunction, wound healing, frailty, and body composition. Essential measurement instruments could not be identified for any outcomes other than mortality timepoints, and studies report a large variability in available instruments [18^▪^,19^▪^]. In CONCISE, suggested instruments included the SF-36 Physical Component Score (SF-36), 30-s sit-to-stand test (30STS), six-minute walk test (6MWT) and short physical performance battery (SPPB) for physical function; the Barthel and Katz indices for activities of daily living (ADLs); the Global Leadership Initiative on Malnutrition (GLIM) criteria for nutritional status and handgrip strength for muscle function. Rigorous reporting of patient-reported outcomes is essential, including transparent handling of missing data patterns, as outlined in the SPIRIT-PRO Extension guidelines [20]. Recent guideline updates have incorporated recommendations from CONCISE, emphasizing the role of physical function tests as primary outcomes in critical care nutrition research [21^▪▪^]. While physical function tests, such as the 6-min walk test and hand grip strength, provide objective assessments of functional capacity, they represent performance-based measures that complement self-reported, patient-centered outcomes.

To standardize reporting of GI function assessment, COSMOGI (core outcome set of daily monitoring of gastrointestinal function) was developed and published in 2024 [17]. Previous research suffered from inconsistent definitions and subjective assessment methods, preventing comparison across studies and limiting the development of evidence-based synthesis. GI dysfunction in critically ill patients is common and associated with higher mortality rates and prolonged ICU stays [15,17,22,23^▪^].

COSMOGI was developed by a large, multinational panel (368 registered participants) of researchers, clinicians, and patient representatives, and consensus was reached on 13 essential daily monitoring instruments for gastrointestinal dysfunction. Traditional COS, like CONCISE, focuses on long-term effectiveness outcomes measured weeks to months after interventions. COSMOGI instead addresses the need for standardized daily monitoring variables during acute illness, allowing for the pooling of standardized data from multiple studies to provide better definitions of GI dysfunction and failure and enable the monitoring of GI treatment effects during trials, including trials assessing enteral nutrition [17]. The 13 essential COSMOGI outcomes consist of signs and symptoms (abdominal distension, bowel dilatation, intra-abdominal pressure, abdominal pain, stool passage, vomiting, GI bleeding), treatment interventions (parenteral nutrition use due to enteral intolerance, prokinetics, postpyloric feeding), and clinical entities (lower GI paralysis, gastroparesis, enteral nutrition intolerance). The panel found consensus on a detailed definition for each outcome, enabling consistent application across diverse study populations. The definitions are directly applicable and should help researchers when developing electronic record forms. Consistent reporting based on COSMOGI should contribute to future refinement of the definition of GI dysfunction and development of new tools for its assessment.

## LIMITATIONS OF CORE OUTCOME SETS

A key issue is feasibility, as some core outcome sets include outcomes and/or measurement instruments that are resource-intensive and impractical to measure in all clinical trials. Concerns regarding feasibility were the main reason so few measurement instruments were mandated in CONCISE. Feasibility issues were also encountered in COSMOGI, where some monitoring outcomes were defined as essential for some patients, but are not assessable in all patients. Feasibility challenges may also contribute to the low uptake of core outcome sets, with only 13% of Cochrane systematic reviews citing an existing core outcome set, and evidence indicating that core outcome sets are rarely used to inform the development of clinical guidelines [24^▪^,25^▪^]. This challenge is further complicated by the separation of outcomes and instruments that are generally voted on in different rounds. While outcomes might be considered essential, i.e., physical function, a measurement instrument or a feasible definition might be lacking. This leads to a discrepancy between what is deemed important and what is achievable in research. For instance, CONCISE failed to achieve consensus on measurement instruments for infection, which was identified as an “essential” outcome [16]. In addition, low participation [26] or difficulty reaching consensus may further challenge the successful completion of consensus processes [27,28].

The quality of systematic reviews informing core outcome sets is variable. A recent evaluation of 175 systematic reviews informing core outcome set development found that the overall quality of these reviews was poor, with 94% of reviews rated as “critically low” or “low” in methodological confidence using AMSTAR 2.0 [A MeaSurement Tool to Assess Systematic Reviews-2] [29^▪▪^]. Limitations in stakeholder representation and methodological consistency are also important concerns. Although both CONCISE and COSMOGI recruited international panels, the stakeholder consultation process had limited geographical representation from certain regions, which may affect the generalizability of the core outcome set. The commonly used modified Delphi methodology is sensitive to the choice of scoring systems, consensus thresholds, and attrition between rounds, which can disproportionately reduce input from certain groups, such as patients and caregivers. In CONCISE, consensus thresholds were altered after the first Delphi round, and high-scoring outcomes were subdivided into “essential” and “recommended” outcomes, an ad hoc change potentially introducing bias. In consensus meetings, dominant professional voices may influence decisions despite earlier anonymous voting.

## TRIALS OF NUTRITION AND METABOLISM INTERVENTION

Since 2010, many randomized controlled trials assessed nutritional interventions in critically ill patients, comparing lower vs. higher energy provision; lower vs. higher protein provision; enteral vs. parenteral nutrition; intermittent vs. continuous enteral nutrition; early vs. delayed EN. Primary and secondary outcomes used in these studies are summarized in Table 1. Taken together, the primary outcome was usually focused on survival or time to discharge alive. Physical function was assessed in a minority of available studies, and sometimes only in selected patients (Table 1). Some studies assessed functional outcome in a separate follow-up publication, though these studies often did not include complete cohorts from the original intention-to-treat populations [30–32]. Funding constraints may limit comprehensive assessments.

**Table 1:** Primary and secondary outcomes in randomized controlled trials of nutritional interventions in critical illness (2010–2025)

Author, year	Comparison; number of patients	Primary outcome	Other outcomes	Main results
Reported some functional outcomes				
Bels 2024 [11^▪▪^]	High vs. standard enteral protein in ICU patients; *n* = 935	EQ-5D-5L score at 30, 90 and 180 days adjusted for baseline score	Time to discharge alive, days alive and at home by day 90, longitudinally measured functional outcomes (6 min walking distance, MRC, handgrip strength)	Worse health-related quality of life in high protein group
Heyland 2022 [10]	Enteral glutamine vs. placebo in patients with severe burns; *n* = 1200	Time to discharge alive	6-month mortality, hospital mortality, LOS, MV duration, gram-negative bacteremia. SF-36 and ADL were assessed in 1/3 of patients	No difference
Chapple 2020 [34]	High vs. standard enteral protein in ICU patients; *n* = 120	Mean daily protein delivery (g/kg/d)	28-day, 90-day mortality, ICU and hospital LOS, ventilator-free days, ICU-free days, vasopressor support, RRT, residence at day 90, EQ-5D-5L at day 90	Higher protein delivery (1.52 vs. 0.99 g/kg/d protein), no difference in clinical outcomes including 90-day mortality or quality of life
Allingstrup 2017 [38]	Early goal-directed (EGD) vs. standard energy and protein in ICU patients; *n* = 199	physical component summary (PCS) score of SF-36 at 6 months	28-day, 90-day and 6-month mortality, LOS, MV, RRT, new organ failure, infections, energy and protein balance, hypo- and hyperglycemia and insulin dose	No difference, except higher energy and protein balance and more hyperglycemia and insulin in the EGD group
Doig 2015 [39]	IV amino acid supplement (up to 100 g/day) vs. standard care in ICU patients; *n* = 474	Duration of renal dysfunction	ICU and hospital mortality, ICU and hospital LOS, MV duration, organ dysfunction, RRT use, RAND-36 General Health and Physical Function, ECOG Performance Status at day 90	No difference in duration of renal dysfunction. Improved eGFR and urine output. No difference in mortality or quality of life
Doig 2013 [40]	Early PN vs. standard of care in ICU patients with contraindications to EN; *n* = 1372	60-day mortality	Quality of life and physical function (RAND-36) at 60 days, organ failures, LOS, MV, RRT, infection, days with antibiotics and pressure ulcer treatment	No difference, except fewer days of MV in the PN group
Casaer 2011 [41,42^▪▪^]	Early vs. late supplemental PN in ICU patients; *n* = 4640	Duration of dependency on ICU	New infection, antibiotics, organ dysfunction and support, LOS, 6-min walking distance and activities of daily life at hospital discharge. Additional 2 year follow-up published separately assessing mortality and nutrition risk score as well as SF-36 physical and mental component scores [42^▪▪^].	Higher likelihood for discharge alive earlier, less infections and cholestasis in the late group; Two-year mortality and physical functioning were similar in both groups.
Did not report any functional outcomes				
Summers 2025 [33^▪▪^]	Augmented vs. usual protein in ICU patients; *n* = 3397	Days free of index hospitalization and alive by day 90	MV duration, LOS, tracheotomy, new RRT, discharge destination	No difference
Panwar 2024 [43^▪^]	Intermittent vs. continuous EN in ICU patients; *n* = 120	GI intolerance	Hospital mortality, MV-free days, EN volume reached	No difference
Heyland 2023 [35]	High vs. standard protein by any route, *n* = 1301	Time-to-discharge-alive from hospital up to 60 days	60-day and hospital mortality, ICU and hospital LOS, duration of MV, nutritional adequacy, re-admission to ICU and hospital.	No difference, subgroup analysis suggested worse outcome in patients with acute kidney injury and high organ failure scores in the high protein group
Reignier 2023 [44]	Low vs. standard energy and protein (any route) in ventilated patients with shock; *n* = 3044	90-day mortality and time to readiness for ICU discharge	28-day, ICU and hospital mortality, ICU and hospital LOS, SOFA score, calories and proteins delivered; body weight, ICU-acquired infection. glucose and insulin, liver enzymes, GI complications	Faster recovery and fewer complications (vomiting, diarrhoea, bowel ischaemia, liver dysfunction) in the low group
Reignier 2018 [45]	Early EN vs. early PN in ventilated patients with shock; *n* = 2410	28-day mortality	90-day, ICU and hospital mortality, ICU and hospital LOS, SOFA score, calories and proteins delivered; body weight, ICU-acquired infection, glucose and insulin, liver enzymes, GI complications	No difference except more GI complications in the EN group
TARGET investigators 2018 [30,46]	Energy-dense vs. routine EN in ICU patients; *n* = 3957	90-day mortality	28-day and in-hospital mortality, ICU- and hospital-free days, days free of organ support, MV, vasopressors, new renal replacement therapy, positive blood cultures and intravenous antimicrobials. A long term follow up [30], not including the entire ITT population, surveyed EuroQol 5D-5L.	No difference Long-term follow up: no difference in occupancy, hours worked or effectiveness at work (*n* = 818), disability (*n* = 1208) or participation in key life activities (*n* = 705).
Rugeles 2016 [47]	High-protein hypocaloric vs. normocaloric EN in ICU patients; *n* = 120	Delta SOFA within 48h	28-day mortality, ΔSOFA at 96 h, insulin requirements, hyper- and hypoglycemia, ICU LOS, MV days	Lower insulin requirements in the hypocaloric group
Petros 2016 [48]	Hypocaloric vs. normocaloric nutrition in ICU patients; *n* = 100	Nosocomial infections during the ICU stay	ICU, hospital, and 28-day mortality, insulin demand, GI intolerance	More infections, less insulin and less GI intolerance in the hypocaloric group
Arabi 2015 [49]	Low vs. standard EN in ICU patients; *n* = 894	90-day mortality	ICU and hospital mortality, 28- and 180-day mortality, SOFA scores, days free from MV and ICU, hospital LOS, hypoglycemia, -kalemia, -magnesemia and phosphatemia, red blood cells transfusions, ICU-associated infections, feeding intolerance (vomiting, abdominal distention, or a GRV >200 ml) and diarrhea.	No difference
Peake 2014 [50,51]	Energy-dense (1.5 kcal/ml) vs. routine (1.0 kcal/ml) EN in mechanically ventilated ICU patients; *n* = 112	Daily calorie delivery from study EN.	90-day mortality, ICU and hospital mortality, ICU and hospital LOS, ventilator-free days, GI tolerance. One-year follow-up (*n* = 39), published separately [50]: SF-36v2 Physical and Mental Component Summary scores, EQ-5D-5L, employment status	46% increase in calorie delivery. Borderline nonsignificant difference in 90-d mortality (20% vs. 37%, *P* = 0.057). One-year follow-up: No difference in quality of life or EQ-5D-5L scores. More patients employed in energy-dense group.
Heyland 2013 [52]	Glutamine, antioxidants, both or placebo in ICU patients; *n* = 1223	28-day mortality	Hospital and 6-month mortality, time to discharge alive from ICU and hospital, ICU and hospital LOS, time to MV discontinuation, SOFA scores, infection rates.	No difference. Subgroup of patients with multiple organ failure showed increased mortality with glutamine.
Heidegger 2013 [36]	Supplemental PN vs. no supplemental PN in patients not reaching energy target after 3 days in the ICU, *n* = 153	Nosocomial infection after intervention (8 days) to day 28	Antibiotic-free days, duration of MV, ICU and hospital LOS until day 28, ICU and 28-day mortality, duration of RRT, insulin, glycemia, CRP, liver tests, phosphatemia, steroids	Less nosocomial infections between days 9 and 28 in the SPN group. More episodes of hyperglycemia in the SPN group
Rice 2012 [31,32,53]	Initial trophic vs. full feeding in mechanically ventilated patients with acute lung injury; *n* = 1000	Ventilator-free days to study day 28	60-day mortality; ICU- and organ failure–free days, daily percentage of goal EN, GI intolerance, new infections Separate follow up trials on cognitive function were published [31,32], assessing 6 min walking distance, cognitive impairment, SF-36, employment status.	No difference except less GI intolerance in the low/trophic group No difference in one year functional outcome analysis.
Arabi 2011 [54]	Permissive underfeeding vs. standard feeding in ICU patients; *n* = 240	28-day mortality	ICU, hospital, and 180-day mortality; ICU and hospital LOS; MV duration, health-care associated infections, RRT, transfusions, hypoglycemia and -kalemia.	Lower hospital mortality in the permissive underfeeding group
Rice 2011 [55]	Initial trophic vs. full feeding in mechanically ventilated patients with acute respiratory failure; *n* = 200	Ventilator-free days to study day 28	28-day and hospital mortality; ICU-, hospital- and organ failure–free days, GI intolerance, new infections	No difference except less GI intolerance in the low/trophic group

Summary of outcomes reported in randomized controlled trials (≥100 patients) comparing nutritional interventions in critically ill adults. Studies are categorized by whether functional outcomes were assessed in the primary publication and ordered by publication year. The table includes trial characteristics (intervention type and sample size), primary outcomes, secondary outcomes, and main findings. Bold text indicates functional outcome measures aligned with CONCISE recommendations. Secondary analyses and follow-up studies, where applicable, are summarized together with original studies.

ADL, activities of daily living; EGD, early goal-directed; EN, enteral nutrition; EQ-5D-5L, EuroQol 5-dimension 5-level questionnaire; GI, gastrointestinal; GRV, gastric residual volume; ICU, intensive care unit; ITT, intention-to-treat; LOS, length of stay; MRC, Medical Research Council scale; MV, mechanical ventilation; PCS, physical component summary; PN, parenteral nutrition; RAND-36, RAND 36-Item Health Survey; RRT, renal replacement therapy; SF-36, Short Form-36 Health Survey; SOFA, Sequential Organ Failure Assessment; SPN, supplemental parenteral nutrition.

In CONCISE, no essential measurement instruments could be identified, partly because few existing studies assessed outcomes after ICU discharge, resulting in insufficient evidence to support their selection [13^▪^,^16^]. However, some more recent trials have started implementing these outcomes. The PRECISe trial, a double-blinded, multicentre trial randomizing 935 patients to receive either high protein (target 2.0 g/kg/day) or standard protein (target 1.3 g/kg/day) enteral nutrition, published in 2024, employed health-related quality of life as the primary endpoint rather than traditional mortality or length of stay measures [11^▪▪^]. Contrary to expectations, patients receiving higher protein demonstrated worse health-related quality of life scores across multiple timepoints extending to 180 days postrandomization. The EFFORT Protein and TARGET Protein trials further reinforced concerns about higher protein delivery, but in contrast to the PRECISe trial, no functional outcomes are reported [33^▪▪^,^35^].

That a nutritional intervention aiming for higher provision of any nutrients during the ICU stay (short time period for most patients) may result in improved survival is unlikely, as confirmed by evidence in RCTs (Table 1). Studies aiming at higher energy, protein, or antioxidant provision early during ICU stay have all shown negligible [36], absent, or adverse effects regarding any outcomes, suggesting potential harm mainly in the subgroups of the most severely ill patients [37^▪^].

Some interventions have also been assessed in systematic reviews. Fuentes Padilla and colleagues compared early enteral nutrition (EN) vs. delayed EN with or without supplemental parenteral nutrition (PN), including seven RCTs with a total of 345 participants [56]. The authors concluded that due to the very low quality of evidence, it is uncertain whether early EN vs. delayed EN or early EN plus supplemental PN vs. delayed EN plus supplemental PN affects outcomes. Outcomes studied in meta-analyses were mortality within 30 days, feeding intolerance, gastrointestinal complications, and pneumonia for the first comparison, and mortality, infectious complications, and duration of mechanical ventilation for the second comparison. Importantly, enteral feeding intolerance, gastrointestinal complications, and infectious complications are not uniformly defined across studies included in meta-analyses [57^▪^,^58^]. Several studies and meta-analyses have reported achievement of enteral calories during ICU stay as an outcome [59^▪^,60^▪^], despite limited patient relevance and the rejection of the hypothesis that greater calorie delivery improves outcomes [37^▪^,61^▪^]. At the same time, it is seldom considered how nutrition is linked to metabolism and GI dysfunction, potentially influencing outcomes [22,62^▪^].

## KNOWLEDGE GAPS AND FUTURE RESEARCH

Nutrition trials often report delivery of calories/protein without assessing metabolic responses (e.g., glucose handling, protein synthesis, substrate oxidation), thereby limiting understanding of how nutrition influences short-term clinical outcomes. Longer-term patient-centered outcomes have been reported inconsistently. Traditional approaches focusing on maximizing early nutrient delivery have proven inadequate, with recent trials demonstrating neutral or harmful effects of higher protein provision. Accordingly, the focus in future studies should be: 1) nutritional intervention other than aiming for higher provision of nutrients early after ICU admission; 2) nutritional intervention initiated during the later phase of illness, possibly after discharge from the ICU; 3) outcomes other than survival; 4) defining metabolic interventions and respective outcome measures. Metabolic interventions remain poorly defined despite inclusion in core outcome frameworks.

The gap between research findings and clinical implementation remains a persistent challenge. Recent research demonstrates that core outcome set adoption remains limited, with many clinicians unaware of standardized measurement recommendations [24^▪^]. Available core outcome sets hope to transform critical care nutrition research by enabling standardized reporting, thus advancing research quality and facilitating evidence synthesis. The emergence of quality-of-life measures as primary endpoints reflects an appropriate focus on patient-centered outcomes. However, adoption remains limited. A critical consideration in core outcome set implementation is maintaining a manageable scope. Core outcome sets must balance comprehensiveness with feasibility; adding new outcomes should prompt consideration of which existing measures might be removed. Practical factors such as measurement methods and participant burden significantly impact completion rates. Remote assessment methods, including telephone or smartphone-based evaluations, can reduce patient burden by eliminating the need for hospital visits.

Muscle ultrasound has emerged as a potential noninvasive approach for quantifying muscle mass changes during critical illness. Recent studies demonstrate that rectus femoris cross-sectional area measured via ultrasound may allow assessment of muscle mass, which is potentially linked to mortality and ICU-acquired weakness [63^▪^,64^▪^]. While muscle thickness assessed by ultrasound may potentially provide valuable information in mechanistic studies exploring nutritional interventions, it represents a surrogate outcome rather than a patient-centered endpoint. This measure may be most appropriate for smaller physiological studies rather than as a primary outcome for all clinical trials. Bioelectrical impedance analysis is another potential assessment tool that could assess changes in body composition during and after critical illness [65^▪^]. However, standardization of measurement protocols remains a priority for widespread clinical implementation. Missing data due to death or early hospital discharge represents an additional challenge in functional outcome assessment.

Future research should assess these novel techniques, incorporate physical function, identify which most adequately represent the recovery trajectory, and aim to develop practical biomarkers and phenotyping tools that enable personalized nutrition interventions [66]. Recent work has emphasized the importance of “readiness for feeding” indicators and personalized approaches to nutrition therapy in critical care [67^▪▪^].

## CONCLUSION

**FIGURE 1:**
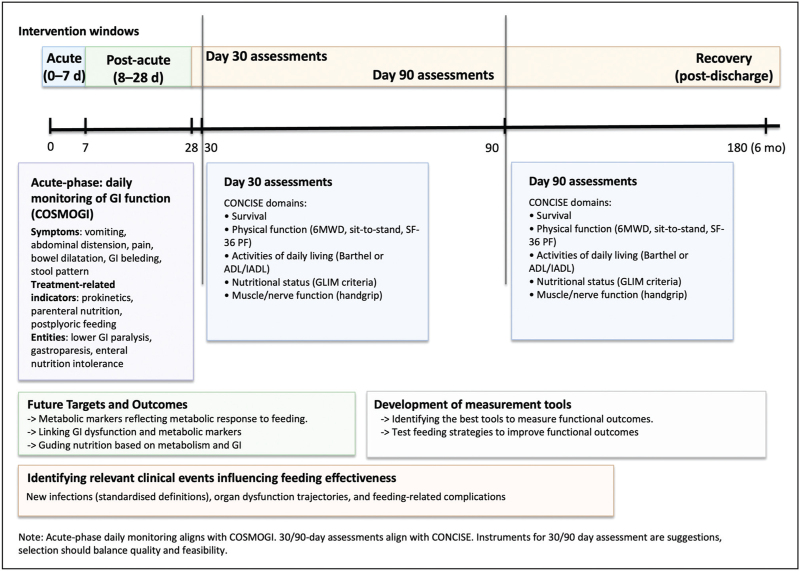
Schematic representation of the timing and domains for outcome assessment in critical care nutrition and metabolism intervention trials based on COSMOGI and CONCISE core outcome sets. During the acute phase, daily monitoring of gastrointestinal function follows COSMOGI recommendations. Standardized assessments at days 30 and 90 postrandomization follow CONCISE recommendations for essential outcomes, including survival, physical function, activities of daily living, nutritional status, and muscle/nerve function. Future research targets include the identification of metabolic response biomarkers, optimizing feeding strategies based on GI dysfunction patterns, and developing and validating measurement tools to assess functional outcomes. 30STS, 30-s sit-to-stand test; 6MWD, Six-minute walk distance; ADL, Activities of daily living; CONCISE, Core Outcome Measures for Clinical Effectiveness Trials of Nutritional and Metabolic Interventions in Critical Illness; COSMOGI, core outcome set of daily monitoring of gastrointestinal function; GI, gastrointestinal; GLIM, Global Leadership Initiative on Malnutrition; IADL, instrumental activities of daily living; SF-36 PF, Short Form-36 Physical Function subscale.

Contemporary randomized trials of nutrition and metabolic care in critical illness have not shown that higher early macronutrient targets improve survival, and emerging evidence suggests potential harm with higher protein delivery in the ICU. These findings indicate that a different approach to interventions and outcomes is needed. The field should now match interventions to outcomes that reflect patient priorities beyond mortality. Assessment at 30 and 90 days according to CONCISE and adopting COSMOGI to structure daily gastrointestinal monitoring should make trials more comparable (Fig. 1), albeit consensus on some measurement instruments is currently lacking. Using clearly defined standardized outcome measures according to the biological plausibility of the effect of intervention, and considering direct or indirect relevance for patients, should be the cornerstone for future research.

## Acknowledgements

*We would like to thank all collaborators and participants of the CONCISE and COSMOGI core outcome sets.*

### Financial support and sponsorship

*The Estonian Research Council (Grant PRG1255) financed the software necessary for the COSMOGI study.*

### Conflicts of interest

*K.F.B. reports no conflicts of interest. A.R.B. is holding a grant from the Estonian Research Council (PRG1255). T.D. reports no conflict of interest.*

## REFERENCES AND RECOMMENDED READING

Papers of particular interest, published within the annual period of review, have been highlighted as:

▪ of special interest
▪▪ of outstanding interest
